# Patient- and Family-centered Rounding: A Single-site Look into the Room

**DOI:** 10.1097/pq9.0000000000000421

**Published:** 2021-06-23

**Authors:** Alexandra Rubin, Rachel R. Osborn, Madeline J. Nowicki, Kira Surber, Jamie L. Rashty, Alanna Shefler, Kelly S. Parent, Kimberly K. Monroe, Kerry P. Mychaliska

**Affiliations:** From the * Department of Pediatrics, The University of Michigan Medical School, C.S. Mott Children’s Hospital, Ann Arbor, Mich.; † Department of Pediatrics, Yale University School of Medicine, New Haven, Conn.; ‡ Department of Social Work, The University of Michigan Medical School, C.S. Mott Children’s Hospital, Ann Arbor, Mich.

## Abstract

**Introduction:**

The American Academy of Pediatrics recommends Patient- and Family-centered Rounds (PFCRs) to improve communication between the healthcare team and families while allowing the latter to participate in medical decision-making. PFCRs have a secondary goal of increasing rounds’ efficiency and providing a positive learning environment for residents and students. There are many published best practices for PFCR. Our study provides an observational evaluation of PFCR in an academic tertiary medical center using a checklist created from such published best practices.

**Methods:**

We created a standardized observation checklist based on published guidelines. Study members observed 200 individual rounding encounters using this instrument. All inpatient, nonsurgical rounding teams in the fall of 2014 were included and analyzed using descriptive statistics.

**Results:**

The average rounding encounter included 9 team members, lasted 9 minutes and 24 seconds, with the medical team entering the patient room for 80.0% of encounters. Families were invited to participate in 60% of the encounters. Lay language was utilized in 62% of the encounters, although 99.5% of the encounters staff used medical terminology. Nursing was present in 64.5% of encounters but presented in only 13.5% of those encounters. The teaching-attending modeled patient interaction behaviors such as eye contact, nodding, and leaning forward in 31%–51% of encounters.

**Conclusions:**

Despite published best practices, medical teams at a large tertiary care center did not adhere to many components of published PCFR guidelines. Future studies should focus on family and physician experience to identify improvement strategies for rounds.

## INTRODUCTION

Patient and family involvement in rounds has become the standard of care in pediatrics. The Institute of Medicine (IOM) Committee on Quality of Health Care in America recommends the following principles to improve patient care: the patient as the source of control, shared knowledge, free flow of information, and cooperation among clinicians.^1^ These concepts have been incorporated in practice by implementing Patient- and Family-centered Rounds (PFCRs). PFCRs are a commonly practiced form of rounding in pediatrics throughout the United States and Canada.^2^ The American Academy of Pediatrics (AAP) describes PFCR as a collaboration among patients, families, physicians, nurses, and other medical team members to support and facilitate choice for the child’s family while sharing honest and unbiased information.^3^

PFCRs have been shown to improve discharge timeliness by reducing hospital workflow inefficiencies and improving patient and family satisfaction with care.^1,2,4–12^ When done well, parents report that PFCR helped them understand team roles more authentically and empowered them to advocate effectively for their children, positively affecting care.^13–16^ However, many authentic barriers to PFCR still exist. Studies have demonstrated learner hesitancy when presenting in front of the family, impaired senior resident autonomy, lack of nurse presence and input on rounds, variable family participation, medical teams spending time talking outside of the room, and many concerns about time and space with large teams in academic centers.^2,5,13,17–20^

In 2007, Muething et al^5^ at the Cincinnati Children’s Hospital Medical Center published several practical and specific recommendations to maximize the benefits of PFCR. Recommendations include entering the patient room and forming a circle inclusive of the patient and family, making eye contact, involving the family in daily plans and discharge goals, digitally entering orders, prescriptions, discharge summaries, or home health care plans when necessary, and asking families for permission before teaching in the patient room.^5^ This study characterizes the frequency to which medical rounding teams adhere to observable published best practices on PFCR.

## METHODS

Although the literature is replete with guidelines regarding PFCR, a consensus paper delineating specific guidelines on the format of PFCR supported by the AAP did not exist at the time of this study. We selected Muething et al’s recommendations because it delineates specific practices, allowing for creating an observational checklist instrument. Subsequent published PFCR literature, 2017 Cox et al,^21^ supports many of the same qualities as Muething’s, such that our checklist continues to reflect current best practices.

**Fig. 1.**
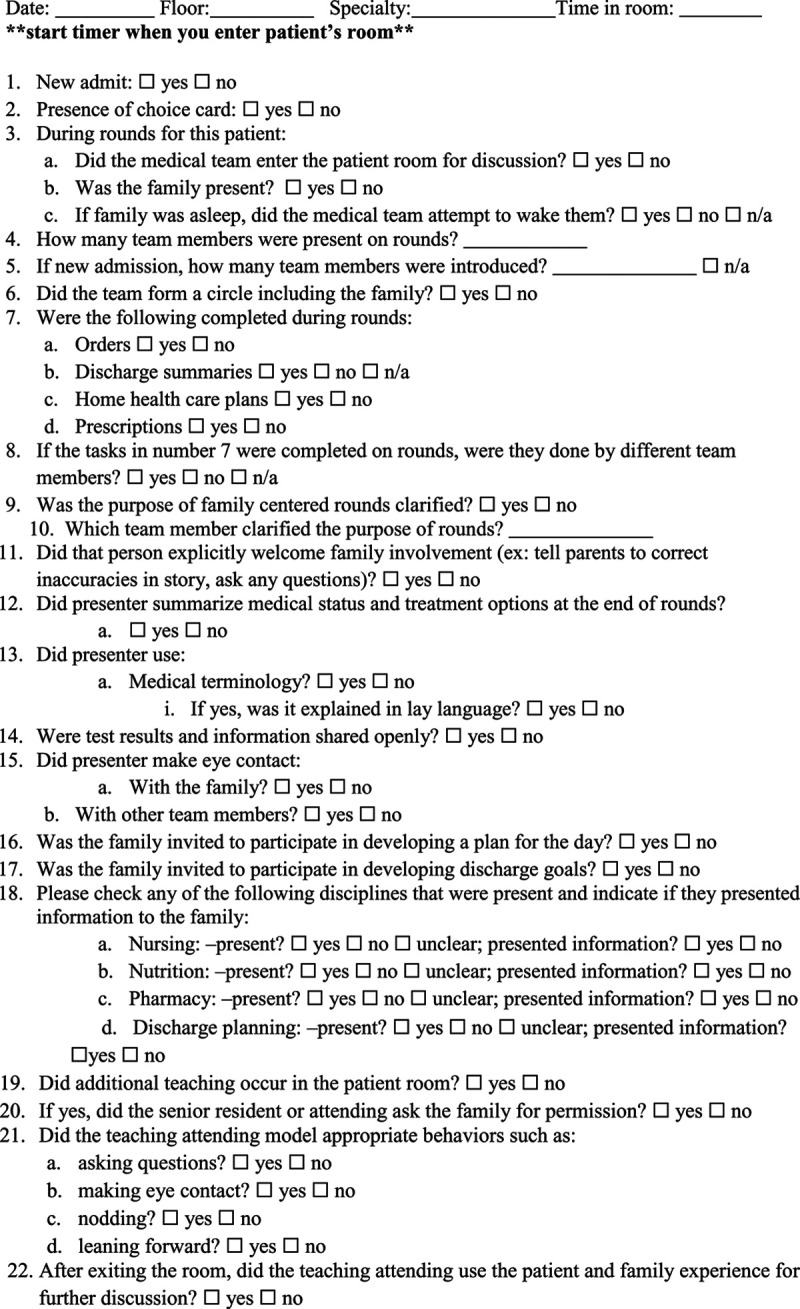
Observational checklist.

All study team members reviewed and edited our checklist to include only observable items, such as family and medical team member presence, completion of tasks, and explicit inclusion of the family in medical decision-making. Before observing rounds, each study team member watched a PFCR video and completed the standardized checklist with responses compared to interrater validity. Further validation of the instrument occurred with the first 41 observations performed by multiple team members to identify areas of uncertainty. Discrepancies were discussed using an iterative process until an agreement about items was reached, and the checklist was finalized to include only readily observable behaviors (Fig. 1).

From October through December of 2014, observations occurred on a random sample of PCFR on general and subspecialty rounding teams at C.S. Mott Children’s Hospital in Ann Arbor, Michigan. We excluded surgical teams from the study. Medical teams were blinded to the study’s purpose to minimize the Hawthorne effect (the alteration of behavior by the subjects of a study due to their awareness of being observed). However, they were explicitly aware of the observer’s presence. Observers did not interact with families. Incomplete observations were excluded from the analysis. Medical providers did not receive prior instruction on PFCR checklist items. Prior nursing staff education on PFCR included encouraging nursing to be present on rounds with expectations to contribute concerns or provide additional information as needed.

We defined PFCR as any encounter in which the patient, family, and multiple, but not all, disciplines (ie, nursing and physicians) were present. Rounds were included if they were in the patient’s room or nearby hallway. For statistical analysis, the checklist instrument items were classified as presenter behavior, team participation, and teaching-attending behavior. All descriptive statistics were performed using Microsoft Excel. The University of Michigan IRB exempted the study.

## RESULTS

During the study period, we included 200 PFCR encounters encompassing 12 pediatric medical subspecialties. We included all encounters that involved the patient and a family member and where multiple disciplines were present. Twenty-seven observations were excluded because of incomplete data; the checklist was not completely filled out. All results are available in Table 1, categorized by presenter behavior, team participation, and teaching-attending behavior.

**Table 1.** Observational Results

Presenter Behavior	No. Encounters Observed (n = 200)
Family was invited to participate in developing discharge goals	81 (40.5%)
Family was invited to participate in developing plan for the day	113 (56.5%)
Presenter summarized medical status and treatment options	116 (58%)
Presenter explicitly welcomed family involvement	120 (60%)
Presenter used lay language	124 (62%)
Presenter made eye contact with the family	137 (68.5%)
Team behavior	
Discharge planning presented information	8 (4%)
Pharmacy presented information	10 (5%)
Nutrition presented information	19 (9.5%)
Nursing presented information	27 (13.5%)
Orders, discharge summaries, and prescriptions were completed	64 (32%)
Home healthcare plans completed during rounds	65 (32.5%)
Discharge summaries completed during round	82 (41%)
Prescriptions completed during round	127 63.5%)
Orders completed during round	131 (65.5%)
Teaching-attending behavior	
After exiting the room, teaching attending used the patient and family experience for further discussion	53 (26.5%)
Teaching attending leaned forward	62 (31%)
Teaching attending nodded	90 (45%)
Teaching attending made eye contact	103 (51.5%)

### Presenter Behavior

The presenter explicitly invited family involvement in 60% (120/200) of encounters and summarized medical status and treatment options at the end of PFCR encounters most of the time (58%, 116/200), including explicit invitations to participate in plan development (56.5%, 113/200). A similar modest majority of encounters utilized lay language in the presentation (62%, 124/200), though nearly every encounter used medical terminology (99.5%, 199/200). Results and information were shared openly in 96.5% (193/200) of encounters.

### Team Participation

The medical team entered the patient room for rounds in 80.0% (160/200) of encounters, with an average of nine team members (physician, nurse, ancillary staff, etc.) present on rounds. The average rounding time per patient was 9.4 minutes (SD = 4.5). Ancillary staff presence varied, but participation in rounds by nonphysician professionals was consistently low. Nursing was present in 64.5% (129/200) of PFCR encounters, and nurses presented in only 13.5% (27/200). Other ancillary professional services were frequently present but infrequently directly involved in rounding, as noted in Table 1.

### Teaching-attending Behavior

The attending physician’s teaching occurred in 29.5% (59/200) of PFCR encounters in the patient room. Of the patient encounters where teaching occurred, 10% (6/59) of them occurred with the family’s permission. The teaching-attending modeled patient interaction behaviors such as eye contact, nodding, and leaning forward varied, ranging from 31% (62/200) of encounters to just over 50% (103/200). The teaching-attending used the family encounter for further discussion after exiting the room in 26.5% (53/200) of encounters observed.

## DISCUSSION

According to the AAP, a family-centered approach to healthcare leads to more successful health outcomes, a more efficient allocation of resources, and improved patient and family satisfaction.^3^ The PFCR model is bedside work-rounding in which the patient and family share in the evaluation and management plan of the patient. Our study demonstrates the variable inclusion of proposed PFCR best practices at a free-standing children’s hospital.

Other studies have demonstrated that parents are consistent in their desire to understand the diagnosis and plan of care for their hospitalized child,^4^ and components of the PFCR model can promote this comprehension.^5^ Our study demonstrates the inconsistent application of these strategies, most notably that medical terminology was used nearly universally. In contrast, lay language was left out of a significant percentage of rounding encounters. Encouraging participation may not effectively counter the overwhelming impact of excluding families with the language used by medical providers.^22^ This highlights the need to consider family-centered rounds as truly family-centered and not merely physician-centered rounds at the bedside. The challenge lies in maintaining provider-to-provider communication efficiency while still effectively encouraging family participation through accessible language. It is notable that the medical teams communicated a summarized medical status and treatment options 58% (116/200) of the time yet many other goals of PFCR were not adequately achieved. This may speak to the medical team’s desire to apply their typical interprofessional communication to the bedside with the hope that parent presence will lead to improved communication. Information sharing in a direct form is already a part of our standardized approach to rounds without the family’s presence. Simply communicating in front of the parents does not change the content of their understanding. It is not enough to “usually” encourage participation. We must encourage participation in our exact communication style.

PFCRs are often touted as a more efficient model of care. However, this can only be true if we achieve the goals of information-sharing and decision-making at the time of rounds. Though consistent with rounding times at other institutions reported in the literature, the rounding times observed were relatively brief, considering the expansive list of tasks hoped to be completed during the encounter. Future studies should prioritize the efficiency of information sharing among clinicians through the EMR to help achieve this task. Also, we may need to reassess our traditional presentations’ format and structure, as recounting a lengthy review of systems or remote differential may be surpassed in prioritization by communication in the room with the patient and family. The presence of nonphysician team members but their lack of participation in the process was also a target for improvement. As a field, healthcare should be creating time and space for their valuable contributions to the team in many circumstances.

Our study data provide the foundation for future quality improvement projects for sites with similar needs. Simultaneous to this study in 2014, our hospital at-large focused more on improving communication with patients and families. Patient education materials were created to help families understand and engage with PFCR,^23^ and a resident-focused PFCR communication simulation curriculum was trialed and shown to be effective.^24^ This later led to a refined intervention involving faculty communication coaches to promote resident PFCR skill development.^25^ More recently, a faculty development series was implemented, and institutional clinical practice guidelines were created.

There are several limitations to this study. Most notably, the study was conducted at a single site, which may limit generalizability. However, a national PRIS (Pediatric Research in Inpatient Settings) study survey on PCFRs by Rubin et al,^26^ showed that only 20% of medical students, residents, or fellows and 38% of attending’s receive any formal training on the PFCR model. This finding may lead to more generalizability as our medical teams also did not receive any formal training on the PCFR model. Also, an observational checklist was used, and though we took steps to promote reliability among observers, some variability in observations of behaviors may exist. This study also took place between October and December, when many patients would have been placed on contact or droplet precautions. Providers may have been wearing gowns and face masks, making it more difficult to observe their behavior and possibly change the interaction in the room.

Additionally, the presence of nursing, pharmacy, nutrition, and discharge planning staff was occasionally unclear as these team members were not always introduced nor presented information. We also did not collect any data on how the family perceived whether they were included. Thus, we may be over or under-estimating the achievement of the goals focused on the patient and family experience.

## CONCLUSIONS

In this study, PFCR did not consistently follow published guidelines, which likely impacts the patient and family experience. Our study shows that presenter behaviors, team participation, and teaching physician behaviors are significant barriers to inclusive PCFR. Trainee education and physician and staff engagement and satisfaction are also negatively impacted by variabilities in PFCR. Despite the lofty goals for this PCFR model, our data shows we fell short of achieving true patient- and family-centered care. Large academic centers may fall short of achieving true patient- and family-centered care without directed education, focusing on accomplishing rounding tasks, and improving communication during PFCR. Our institutional experience provides support for the need for such education. Future studies and quality improvement projects with focused interventions are needed to further improve patient, family, staff engagement, and participation in PCFR. This effort will allow for a restructuring of the valuable time we spend in rooms with families, improving patient and family satisfaction, patient safety, physician job satisfaction, and trainee education.

## DISCLOSURE

The authors have no financial interest to declare in relation to the content of this article.

## ACKNOWLEDGMENTS

Elizabeth Hill, Priyanka Rao, Courtney Palka, Mayya Malakh, and all the University of Michigan clinicians who assisted with this study.
